# The Comparative Study on the Rapid Decolorization of Azo, Anthraquinone and Triphenylmethane Dyes by Anaerobic Sludge

**DOI:** 10.3390/ijerph13111053

**Published:** 2016-10-28

**Authors:** Daizong Cui, Hao Zhang, Rubao He, Min Zhao

**Affiliations:** College of Life Science, Northeast Forestry University, Harbin 150040, China; echoblack2016@163.com (D.C.); fmswd@163.com (H.Z.); m82191513@163.com (R.H.)

**Keywords:** anaerobic sludge, synthetic dyes, decolorization, biodegradation, microbial community, intermediates

## Abstract

An anaerobic sludge (AS), capable of decolorizing a variety of synthetic dyes, was acclimated and is reported here. The sludge presented a much better dye decolorizing ability than that of different individual strains. A broad spectrum of dyes could be decolorized by the sludge. Continuous decolorization tests showed that the sludge exhibited the ability to decolorize repeated additions of dye. The chemical oxygen demand (COD) removal rate of the dye wastewater reached 52% after 12 h of incubation. Polymerase chain reaction and denaturing gradient gel electrophoresis (PCR-DGGE) profiles revealed that the microbial community changed as a result of varying initial concentrations of dyes. Phylogenetic analysis indicated that microbial populations in the sludge belonged to the phyla Acidobacteria, Firmicutes, Bacteroidetes, Chloroflexi and Proteobacteria. The degradation products of the three types of dye were identified. For azo dyes, the anaerobic sludge converted Methyl Orange to *N*,*N*-dimethylbenzene-1,4-diamine and 4-aminobenzenesulfonic acid; for triphenylmethane dyes, after Malachite Green was decolorized, the analyzed products were found to be a mixture of *N*,*N*-dimethylbenzenamine, 3-dimethyl-aminophenol and 4-dimethylaminobenzophenone; for anthraquinone dyes, two products (acetophenone and 2-methylbenzoic acid) were observed after Reactive Blue 19 decolorization. Together, these results suggest that the anaerobic sludge has promising potential for use in the treatment of industrial wastewater containing various types of dyes.

## 1. Introduction

Synthetic dyes, classified on the basis of their chromophores as azo, anthraquinone, triphenylmethane and heterocyclic dyes, have been extensively used in the textile, printing, paper, cosmetics and food industries. Annually, approximately 2.8 × 10^5^ t of textile wastewater with dyes is discharged into the environment worldwide [1]. These dyes are considered to be relatively persistent pollutants because they are extremely stable when exposed to light and aerobic conditions. Moreover, most dyes are toxic, and their transformation products are carcinogenic and mutagenic [2]. Thus, dye wastes must be treated before they are released into the environment.

Decolorization of industrial dyes can be achieved through physico-chemical methods, such as adsorption, coagulation, precipitation and chemical oxidation. However, these methods have not been widely applied because of their low efficiency, high cost and intensive energy requirements. In recent years, many new processes for dye decolorization have been developed. One promising strategy is to use microbes to decolorize dyes. The biodegradation of dyes is considered to be an environmentally friendly and cost-effective option [3].

At present, a number of studies have focused on the utilization of bacteria to decolorize synthetic dyes. Bacteria are capable of reducing azo dyes under both anaerobic and aerobic conditions; such bacteria, include obligate anaerobes such as *Clostridium* sp. [4], facultative anaerobes such as *Enterobacter* sp. and *Escherichia* sp. [5,6] and some aerobes such as *Pseudomonas* sp. [7]. Similarly, several phylogenetically diverse bacteria, such as *Shewanella* sp., *Citrobacter* sp. and *Staphylococcus* sp., can decolorize anthraquinone or triphenylmethane dyes [8,9,10]. Although synthetic dyes can be decolorized by different microorganisms, there is little information about a single stain that is able to reduce a broad range of dyes. Thus, the use of a specific strain on dye decolorization is not practical in treating textile wastewater. It has been reported that treatment systems with mixed microbial populations are more effective in decolorization of different dyes than those with single strains. After acclimatization, the mixed cultures, which are composed of stable microbial pellets, may have a strong ability to decolorize synthetic dyes. Moreover, the high microbial diversity and the synergistic metabolic activities of the mixed consortia promote a higher degree of dye decolorization. Therefore, an acclimated microbial community is a more appropriate and efficient approach to the decolorization of different types of dyes.

In this study, we report the investigation of a novel anaerobic sludge capable of decolorizing several classes of textile dyes. To obtain more details about the anaerobic decolorization process, the decolorization characteristics were studied. The dynamic composition of the microbial community of the anaerobic sludge was assessed by PCR-DGGE. Gas chromatography-mass spectrometry (GC-MS) and liquid chromatography-mass spectrometry (LC-MS) were used to determine the intermediate products after dye decolorization. All the results showed that the anaerobic sludge has a high ability to decolorize azo, anthraquinone and triphenylmethane dyes and has a favorable potential for treatment of textile wastewater.

## 2. Materials and Methods

### 2.1. Dyes and Chemicals

Three typical synthetic dyes (Methyl Orange, Reactive Blue 19 and Malachite Green) were used for the anaerobic sludge acclimation. Fifteen dyes (containing five each of azo, anthraquinone and triphenylmethane dyes) were used to monitor the decolorization abilities of the developed anaerobic sludge. All of the dyes were procured from the Guangfu Fine Chemical Research Institute (Tianjin, China). The specifications of each dye are given in Table 1. The rTaq DNA polymerase, PCR purification kit, pMD-18T vector and *E. coli* JM109 were purchased from TaKaRa Biotechnology Co., Ltd. (Dalian, China). All other chemicals were of analytical grade or the highest quality.

### 2.2. Inoculum, Basal Medium Characteristics and the Anaerobic Reactor

Anaerobic sludge was collected from an anaerobic digester located at the Wenchang wastewater treatment plant in Harbin (Heilongjiang Province, China). The sludge was originally developed for treatment of a largely domestic effluent. A basal medium of the following composition was used: 5 g·L^−1^ glucose, 2 g·L^−1^ NH_4_Cl, 1.5 g·L^−1^ Na_2_HPO_4_, 1 g·L^−1^ MgSO_4_·7H_2_O, 0.5 g·L^−1^ KCl. The anaerobic reactor used in this study was a PVC vessel with a capacity of 10 L, equipped with a mechanical stirrer to provide a stable mixture of substrate and sludge. The initial sludge concentration was 5 g mixed liquor suspended solids (MLSS) per liter. The temperature was kept at 25 °C and the pH was maintained at 7.0. During the operation process, the dissolved oxygen concentration (DO) was controlled to between 0–0.3 mg·L^−1^.

### 2.3. Experimental Setup and Operation

The anaerobic reactors was operated in successive 24 h cycles consisted of a filling phase of 30 min, a reaction phase of 21.5 h, a settling phase of 1 h, a draw phase of 45 min and an idle time of 15 min. To acclimatize the sludge, the reactor was initially operated with dye-free medium for one month. Then during ten days, medium containing fresh dye was added into the reactor (containing 50 mg·L^−1^ Methyl Orange, Reactive Blue 19 and Malachite Green, respectively). During the next fifty days, the dye concentration was gradually increased to 300 mg·L^−1^ for the three dyes mentioned above (increasing 50 mg·L^−1^ of each dye for every ten days). After the stabilization of the feed solution, the reactors were operated for 30 days. Sludge samples were collected at the last operation day for each dye concentrations (0–300 mg·L^−1^). The pellets were washed twice with 50 mmol·L^−1^ phosphate buffer (pH 7.0) and stored at −20 °C for molecular analysis.

### 2.4. Semi-Continuous Study

After sludge acclimatization, a semi-continuous decolorization test was conducted to indicate whether long-term degradation of dyes could be stably attained. The system was operated at a hydraulic retention time (HRT) of 24 h which we mentioned above. The anaerobic system was fed with 100 mg·L^−1^ of Methyl Orange, Reactive Blue 19 and Malachite Green for ten days, respectively. The decolorization rate for each dye was measured. During the treatment process, wastewater containing 100 mg·L^−1^ Methyl Orange, Reactive Blue 19 and Malachite Green was incubated with the sludge to detect the COD removal rate.

### 2.5. Batch Assays

#### 2.5.1. Decolorization of Different Dyes by the Anaerobic Sludge

Batch tests were conducted to determine if the anaerobic sludge had the ability to decolorize synthetic dyes with different chemical structures. Serum bottles (70 mL) were filled with 50 mL of distilled water with the a dye concentration of 100 mg·L^−1^. The bottles were flushed with nitrogen gas for 10 min to remove oxygen and sealed with rubber stoppers. Each bottle was inoculated with 150 mg (cell dry weight) of anaerobic sludge. The bottles were incubated in a temperature-controlled cabinet maintained at 37 °C. Decolorization assays were carried out after a 12 h incubation. All assays were performed in triplicate.

#### 2.5.2. Individual Strains vs. Anaerobic Sludge

Three individual strains (*Escherichia coli* CD-2, *Klebsiella pneumoniae* Y3 and *Bacillus subtilis* WD23) and their mixtures were used for the decolorization comparison with the anaerobic sludge. Methyl Orange, Reactive Blue 19 and Malachite Green were chosen as model dyes for azo, anthraquinone and triphenylmethane dyes, respectively. All three strains were isolated by us from environmental samples and were proven in our previous studies to have the ability to decolorize dyes with different structures [11,12,13].

The three pure cultures were grown aerobically until the OD_600_ reached 1.2. Cells were harvested by centrifugation (10,000 *g*, 3 min), washed twice with sodium phosphate buffer (50 mmol·L^−1^, pH 7.0), transferred to serum bottles and resuspended in 50 mL distilled water to 3 g·L^−1^ (cell dry weight). Moreover, the three strains were mixed equally to make a microbial consortium. Bottles which were inoculated with 3 g·L^−1^ mixed culture and anaerobic sludge are also used in the decolorization tests. Decolorization studies were started by addition of 100 mg·L^−1^ different dyes under anaerobic conditions. Decolorization assays were carried out after a 12 h incubation. All assays were performed in triplicate.

### 2.6. Analytical Methods

The developed sludge was used to monitor the decolorization of different types of dyes under anaerobic conditions. Samples were taken at different time intervals and analyzed for dye decolorization. The dye decolorization rate was estimated by measuring the absorbance at the respective λ_max_ of the different dyes with a UV-vis spectrophotometer (UV-2800, Unico Instruments Co., Ltd., Shanghai, China). The decolorizing activity was calculated using the following equation:

(1) $$\text{Decolorization rate }(\%)=\frac{\left(A_{i}-A_{f}\right)}{A_{i}}\times 100\%$$

where *A_i_* = initial absorbance of dyes, *A_f_* = absorbance of dyes after decolorization. COD and MLSS analyses were carried out according to the methods outlined in the Standard Methods for Examination of Water and Wastewater [14].

### 2.7. Enzymatic Assay

The sludge sample was washed twice with 50 mmol·L^−1^ phosphate buffer (pH 7.0). The sample was resuspended in 10 mL of the same buffer. Cells were disrupted by sonication at 4 °C (5 s, 80% output, 50×). Cell debris was removed by centrifugation for 20 min at 10,000 *g*. The supernatant was used as the crude enzyme.

The azoreductase activity assay system comprised 50 mmol·L^−1^ phosphate buffer (pH 7.0), 100 µmol·L^−1^ NADH, 50 µmol·L^−1^ Methyl Orange, and a suitable amount of crude enzyme. The reaction was initiated by the addition of NADH. Azoreductase activity was detected by following the disappearance of Methyl Orange at its maximum wavelength.

### 2.8. PCR-DGGE Analysis

#### 2.8.1. DNA Extraction and PCR Amplification of the 16S rDNA V3 Region

Sludge samples that acclimated at different periods were collected by centrifugation at 6000 g·min^−1^ for 10 min. Genomic DNA was extracted according to the method described by Lakay et al. [15]. PCR amplification was performed using the forward primer F357-GC(5′-CGCCCGCCGCGCGCGGCGGGCGGGGCGGGGGCACGGGGGGCCTACGGGAGGCAGCAG-3′) and reverse primer R518 (5′-ATTACCGCGGCTGCTGG-3′). The PCR amplification included initial denaturation at 94 °C for 5 min; 10 cycles of 94 °C for 1 min; 60 °C for 1 min and 1 min at 72 °C and 20 cycles of 1 min at 94 °C; 1 min at 50 °C and 1 min at 72 °C followed by a final extension of 10 min at 72 °C. The amplified PCR products were visualized using 1% (w/v) agarose gel-electrophoresis.

#### 2.8.2. DGGE Analysis

DGGE was performed using a Dcode universal mutation detection system (Bio-Rad Inc., Hercules, CA, USA). Samples containing approximately equal amounts of PCR amplification products were loaded on a 10% poly-acrylamide gel in TAE buffer with a denaturing gradient ranging from 30% to 60% of the denaturant (denaturation of 100% corresponded to 7 mol·L^−1^ urea and 40% (v/v) formamide). DGGE was performed at 60 °C with a constant voltage of 160 V for 6 h, by using a gradient delivery system (Model 475, Bio-Rad Inc., Hercules, CA, USA. The gel was then stained by AgNO_3_ and the images of the gel were captured by a bio-image system (Bio-Rad).

DGGE profiles were analyzed using the software Quantity One 4.6.2 (Bio-Rad Inc., Hercules, CA, USA). Dendrograms relating band pattern similarities were automatically calculated using the unweighted pair group method with the arithmetic average (UPGMA) clustering algorithm. UPGMA employs a sequential clustering algorithm, in which local topological relationships are identified in order of similarity, and the phylogenetic tree is built in a stepwise manner. Relative band densities, which were necessary to determine the Shannon diversity index (SDI), were quantified, and the statistical data were exported for further SDI analyses.

#### 2.8.3. Sequencing and Phylogenetic Analysis

Bands of interest were cut out from the DGGE gel, mixed with 20 mL of deionized water, and incubated at 50 °C for 1 h. After centrifugation at 7000 *g* for 3 min, the supernatant was collected and used as a PCR template. The DNA fragment was reamplified using the primers R518 and F357 without GC-clamps. The amplification process was as follows: initial denaturation at 94 °C for 3 min and 30 cycles of denaturation at 94 °C for 30 s, annealing at 54 °C for 1 min and extension at 72 °C for 30 s, followed by a final extension at 72 °C for 10 min. The PCR products were purified with a gel extraction kit and cloned into *E. coli* plasmids for sequencing with a PMD 18-T cloning kit, per the manufacturer’s instructions. The DNA sequences were determined using the chain-termination method with an ABI 3730 DNA sequencer by a commercial service provided by Shanghai Shangon Biological Technology Company (Shanghai, China). Analysis of the sequences consisted of BLAST searches that were used to identify the closest related species within the database.

### 2.9. Analysis of Metabolites Formed after Decolorization of Three Types of Dye

#### 2.9.1. Samples Preparation

One hundred mg·L^−1^ of Methyl Orange, Reactive Blue 19 and Malachite Green were incubated with the anaerobic sludge for 24 h, respectively. After decolorization, the degradation mixtures of three types of dye were centrifuged at 6000 *g* for 10 min. The metabolites in the supernatants were extracted with an equal volume of ethyl acetate, dried over anhydrous Na_2_SO_4_ and evaporated to dryness in a rotary evaporator. The final extracts were dissolved in a small volume of methyl alcohol and then filtered through a 0.22 μm membrane.

#### 2.9.2. GC-MS Analysis

GC-MS analysis of metabolites was performed using an HP-5MS column on a GC/MS system (7800A-7900B; Agilent Technologies Inc., Santa Clara, CA, USA). Helium was used as the carrier gas at a flow rate of 1 mL·min^−1^. The injector temperature was maintained at 280 °C with oven conditions as follows: 60 °C kept constant for 2 min, increased up to 280 °C at 10 °C min^−1^. The compounds were identified on the basis of mass spectra and the NIST library.

#### 2.9.3. LC-MS Analysis

LC-MS analysis was performed using a Thermo LC-MS system (Thermo Fisher Scientific, Waltham, MA, USA). A capillary column Terra C-18 (5 μm × 100 mm length) was used for separation of product intermediates. The mobile phase was a mixture of methyl alcohol and water (50:50, v/v) filtered through a Millipore syringe filter of 0.22 μm. The flow rate of eluent was 0.8 mL·min^−1^, and the injection volume was 20 μL. MS analysis was performed on a mass spectrometer, and the parameters were as follows: spray voltage, 3.5 kV; capillary temperature, 325 °C; capillary voltage, 50 V, tube lens 120 V. The mass range was 50–600 m/z.

## 3. Results

### 3.1. Decolorization Studies

#### 3.1.1. Decolorization of Different Dyes by the Anaerobic Sludge

**Figure 1:**
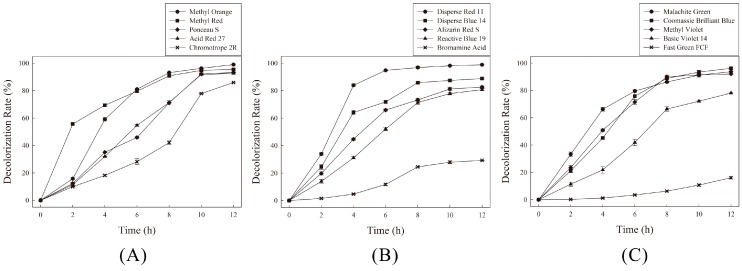
Decolorization of different azo, anthraquinone and triphenylmethane dyes (100 mg·L^−1^) by the anaerobic sludge. (**A**) azo dyes; (**B**) anthraquinone dyes; (**C**) triphenylmethane dyes.

Decolorization of various azo, anthraquinone and triphenylmethane dyes by the anaerobic sludge is shown in Figure 1. The sludge showed a higher decolorization rate for azo dyes, compared with anthraquinone and triphenylmethane dyes. In the case of the azo dyes tested, the sludge exhibited the highest decolorization rate against Methyl Orange. More than 90% of Methyl Orange was decolorized after an 8 h incubation, and the dye was nearly completely decolorized within 12 h.

All other azo dyes were also decolorized effectively by the anaerobic sludge. However, when we used Methyl Orange as the electron acceptor substrate, none of azoreductase activity could be detected in the cytoplasmic extracts. The ability of the sludge to decolorize anthraquinone and triphenylmethane dyes was also tested. The results showed that the maximum percentage of decolorization and the decolorization time varied among the different dyes. For anthraquinone dyes, almost 100% of Disperse Red 11 was decolorized during incubation, and over 80% of Disperse Blue 14, Alizarin Red S and Reactive Blue 19 were decolorized within 12 h. However, only 30% of Bromamine Acid was decolorized within 12 h. For triphenylmethane dyes, the anaerobic sludge decolorized most of the Malachite Green, Coomassie Brilliant Blue, Methyl Violet and Basic Violet 14 in a short time. However, Fast Green FCF was resistant to biodegradation, and only a 15% decolorization rate was achieved after a 12 h incubation.

#### 3.1.2. Individual Strains vs. the Anaerobic Sludge

**Figure 2:**
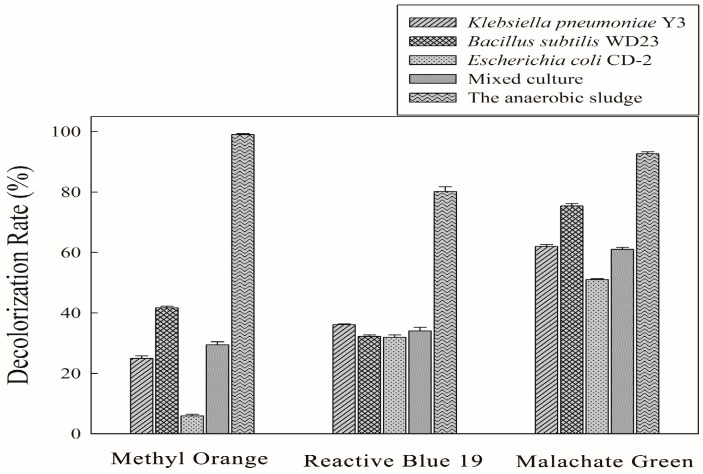
Decolorization performance of Methyl Orange, Reactive Blue 19 and Malachite Green (100 mg·L^−1^) using pure strains, mixed culture and the anaerobic sludge.

Decolorization of model dyes with individual strains and mixed cultures as well as with anaerobic sludge was studied and the results are reported in Figure 2. After a 12 h of incubation, the sludge effective decolorized the three dyes, and the decolorization rates for Methyl Orange, Reactive Blue 19 and Malachite Green were 99%, 80% and 92%, respectively. *K. pneumoniae* Y3 and *E. coli* CD-2 had poor ability to decolorize Methyl Orange in a short time; only 25% and 5% of the dye, respectively, was decolorized during the incubation time. *B. subtilis* WD23 decolorized 75% of the Malachite Green within 12 h. However, less than 50% of the Methyl Orange and Reactive Blue 19 were decolorized during the treatment process. In this study, the three strains were mixed equally to make a microbial consortium. However, the decolorization rate of the mixed culture was not significantly higher by comparison with the three individual strains. We observed that 61% of Malachite Green could be decolorized within 12 h by the mixed culture. However, for Methyl Orange and Reactive Blue 19, only 29% and 34% decolorization rate was attained within 12 h, respectively. The results proved that the anaerobic sludge exhibited a high decolorization efficiency to the three types of dye. After the incubation, all the test dyes could be decolorized over 80%. However, none of the individual strains or the microbial consortium had a high decolorization efficiency for all three dyes. These results showed that the dye decolorization ability of the anaerobic sludge was much better than that of the individual strains.

#### 3.1.3. Decolorization of Dyes by the Anaerobic Sludge under Semi-Continuous Conditions

In this study, the anaerobic reactor was fed 100 mg·L^−1^ of Methyl Orange, Reactive Blue 19 and Malachite Green for ten days, respectively. The decolorization rate of the three dyes was detected during the entire operation process, and the results are shown in Table 2. The results indicated that it was feasible to achieve highly efficient continuous decolorization of Methyl Orange and Malachite Green by decolorization with the anaerobic sludge. The decolorization rates of the two dyes were maintained at up to 95% and 89%, respectively. Moreover, no marked differences in the decolorization efficiency were found during the entire operation process. Despite showing minor instability, the anaerobic sludge decolorized more than 60% of Reactive Blue 19 in every treatment cycle. The anaerobic sludge exhibited an ability to decolorize repeated additions of dye, thus showing that it can be used in multiple cycles of decolorization.

**Figure 3:**
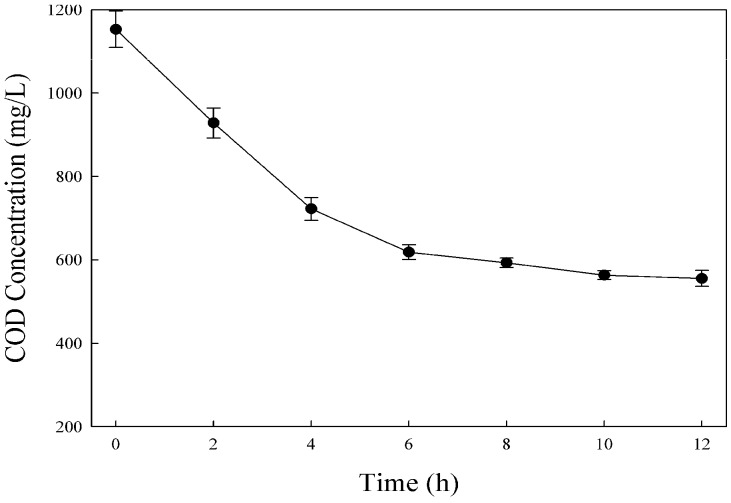
Change of COD during the decolorization of dyes by the anaerobic sludge.

The COD removal rate was detected during the wastewater treatment process. As shown in Figure 3, the COD removal efficiency was high at the beginning of the reaction. After 6 h, the COD decreased from 1150 to 618 mg·L^−1^, and the removal rate was approximately 46%. However, the COD was nearly unchanged in the second 6 h incubation. Finally, the COD decreased to 555 mg·L^−1^ after a 12 h incubation, and a longer treatment process did not produce further COD reduction.

### 3.2. Dye Decolorization Microbial Community

**Figure 4:**
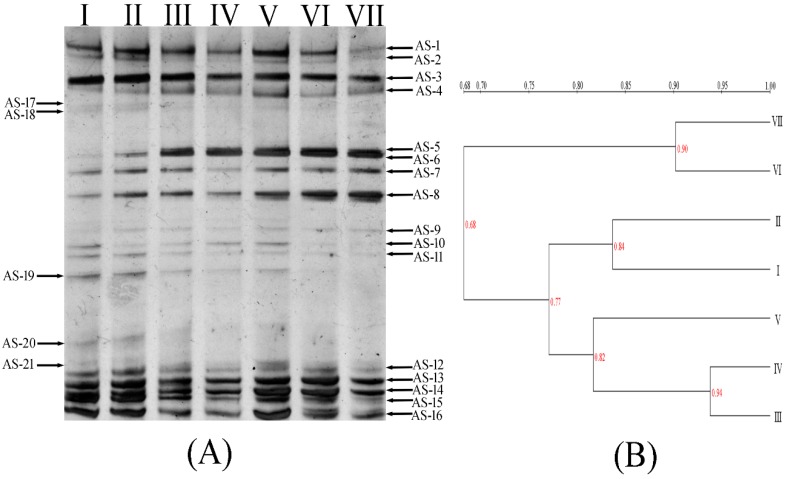
DGGE fingerprints of microbial communities (**A**) and cluster analysis based on “UPGMA” method (**B**) in the samples at different stages of the anaerobic system running. Lane designations: I: original microbial community; II: microbial samples for continuously decolorizing 50 mg·L^−1^ of three model dyes (Methyl Orange, Reactive Blue 19 and Malachite Green) after 10 days; III: microbial samples for continuously decolorizing 100 mg·L^−1^ of three model dyes after 10 days; IV: microbial samples for continuously decolorizing 150 mg·L^−1^ of three model dyes after 10 days; V: microbial samples for continuously decolorizing 200 mg·L^−1^ of three model dyes after 10 days; VI: microbial samples for continuously decolorizing 250 mg·L^−1^ of three model dyes after 10 days; VII: microbial samples for continuously decolorizing 300 mg·L^−1^ of three model dyes after 10 days.

During the sludge acclimation process, the composition of the microbial community was expected to change because some species would be enriched under the new conditions containing different dyes that might be toxic or inhibitory to originally dominant species [16]. In our study, PCR-DGGE analysis of the anaerobic sludge was performed to provide further insight into the microbial diversity. The DGGE fingerprints are shown in Figure 4A. Different band patterns in each lane were observed from the fingerprints, thus revealing that the microbial composition was changed during the acclimation process. With increasing dye concentration, some bands (AS-17, -18, -19, -20, etc.) that were initially present in the medium faded away, whereas some bands (AS-3, -7, -13, -14, etc.) appeared in all the samples. Moreover, some bands emerged and even became more prominent (AS-6, -8, etc.).

The UPGMA clustering analysis was used to analyze the community similarity after exposure different initial dye concentrations. As shown in Figure 4B, populations in sludge with different dye concentrations were categorized into two separate groups. The first group represented the sample I (with no dye addition) and the samples which were acclimated by three types of dye with low concentrations (lower than 200 mg·L^−1^ of each dye, sample II to sample V). The second group represented the sludge were acclimated by high concentrations of dye (higher than 250 mg·L^−1^ of each dye, sample VI to sample VII). The results indicated that the addition of dyes imposed selective pressure on the microbial community. With the increased dye concentrations, the community compositions were also altered. However, the microbial community reached a relatively stable stage when the dye concentration reached 250 mg·L^−1^, as indicated by the high similarity of microbial community structures between the last two lanes, thus suggesting that the anaerobic sludge ultimately adapted to the dye containing environment, and the structure of the bacterial populations in the sludge was maintained.

The fingerprints were also analyzed using the SDI which describes the change in the species dominance of a bacterial community. The SDI for lane I and VII decreased from 3.00 to 2.75, a result suggesting that some microorganisms did not adapt to the environment containing high concentrations of dyes, and they gradually disappeared from the sludge, and thus, the diversity of the bacterial community decreased.

To provide further insight into the microbial diversity, dominant bands were excised and sequenced (Table 3). The identified sequences have been deposited in GenBank under accession numbers KR066383-KR066398. The results of 16S rDNA gene sequencing revealed the presence of an ample diversity of phylotypes. The population of the anaerobic sludge was assigned to five groups, comprising Acidobacteria, Firmicutes (*Lactococcus* sp., *Clostridium* sp. and *Bacillus* sp.), Bacteroidetes (*Prevotella* sp. and *Bacteroides* sp.), Chloroflexi (*Longilinea* sp.) and Proteobacteria (*Klebsiella* sp.).

In the Firmicutes group, three bacteria clones (AS-3, -5, -6) belonged to *Lactococcus* sp. Moreover, the bacteria clones AS-8 and AS-14 presented high sequence similarity to *Clostridium* sp. and *Bacillus* sp., respectively. In the Bacteroidetes group, clone AS-7 had 98% sequence similarity to *Prevotella paludivivens*. The clone AS-12 had the highest similarity to *Bacteroides*. The clone AS-4 had the closest relationship with *Longilinea*, which are filamentous strict anaerobes that ferment carbohydrates. It belongs to the class Anaerolineae and is usually isolated from thermophilic or mesophilic sludge. Clone AS-9 belonged to *Klebsiella*, which is a genus of Gram-negative, facultatively anaerobic bacteria of the family Enterobacteriaceae.

According to the relative intensities of bands on DGGE gel, the dominant band on the line Ⅶ were AS-3, -5, -6, -7, -8, -13, -14, -16. The relative intensity for each band was 7.67%, 7.61%, 7.37%, 7.00%, 13.86%, 8.19%, 8.18% and 5.17%, respectively. Among them, three clones (AS-3, -5, -6) belonged to *Lactococcus* sp.; the clone AS-7 had close relationship with *Prevotella* sp.; the clone AS-8 had the highest similarity to *Clostridium* sp.; the clone AS-14 belonged to *Bacillus* sp.

### 3.3. Monitoring of Intermediates through Anaerobic Degradation

#### 3.3.1. Monitoring of Intermediates after Methyl Orange Degradation

**Figure 5:**
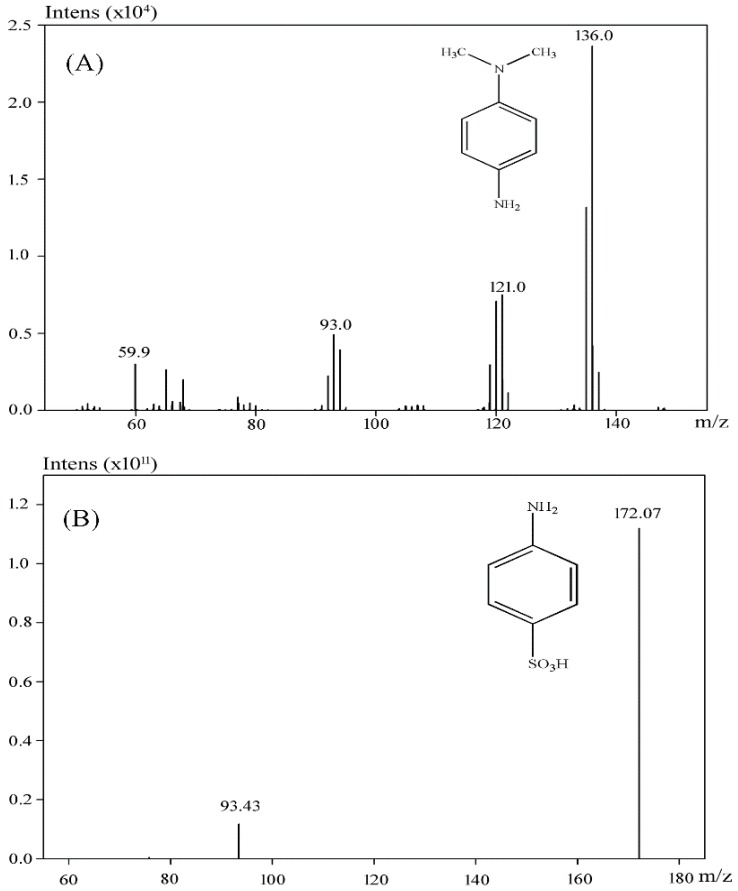
Identification of metabolites of Methyl Orange by GC-MS (**A**) and LC-MS (**B**) analysis.

The reduction products of Methyl Orange were collected for GC-MS analysis. As it shown in Figure 5A, the compound at retention time 19.17 min was identified as *N*,*N*-dimethylbenzene-1,4-diamine. However, the boiling point of 4-aminobenzenesulfonic acid exceeded the temperature limit of the GC-MS, therefore only *N*,*N*-dimethylbenzene-1,4-diamine of the products was detected in the GC chromatogram. To test whether 4-aminobenzenesulfonic acid was present in the degradation mixture, LC-MS was used for further analysis. As shown in Figure 5B, 4-aminobenzenesulfonic acid was identified as one of the Methyl Orange reduction products.

#### 3.3.2. Monitoring of Intermediates after Malachite GreenDegradation

**Figure 6:**
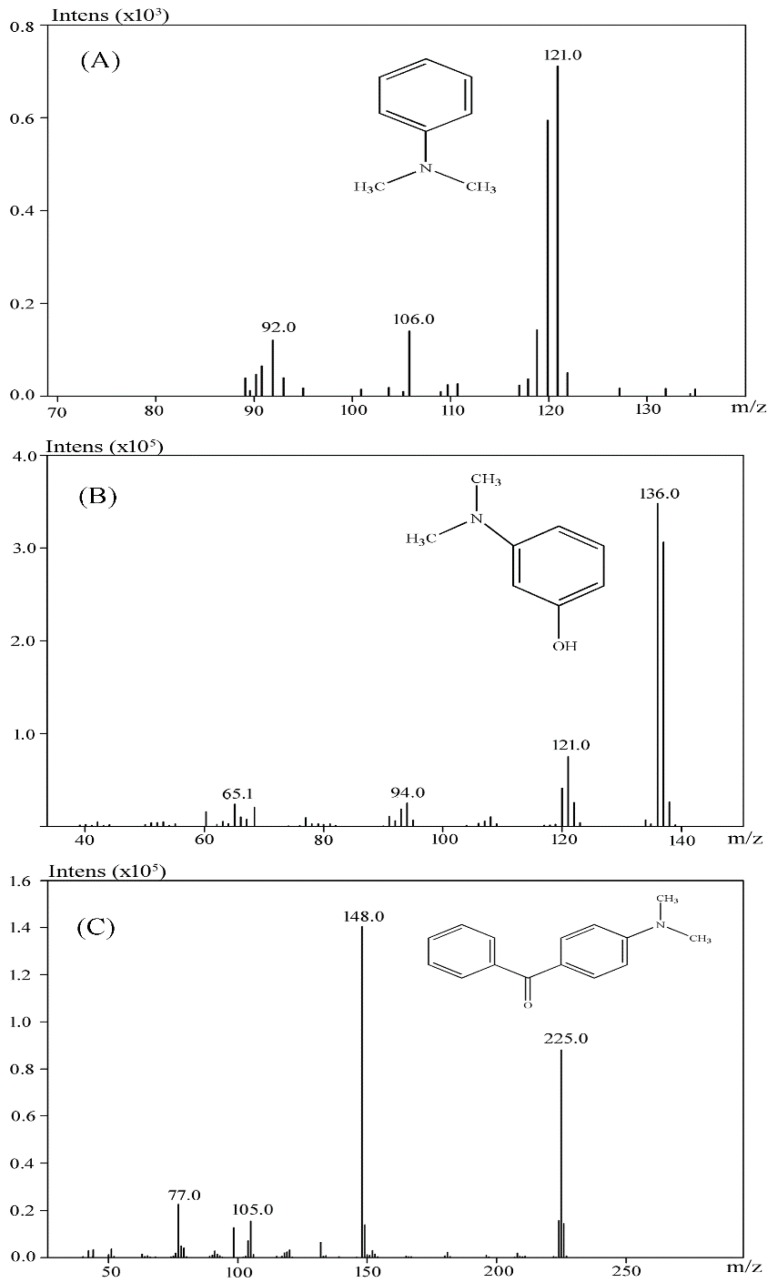
Identification of metabolites of Malachite Green by GC-MS analysis. (**A**) *N*,*N*-dimethyl-benzenamine (retention time 18.73 min); (**B**) 3-dimethylamino-phenol (retention time 19.06 min); (**C**) 4-dimethylaminobenzophenone (retention time 37.71 min).

When the extracted metabolites of Malachite Green were injected into the GC-MS, the mass spectrum identified three products with retention times 18.73, 19.06 and 37.71 min and m/z ratios of 121, 137 and 225, respectively (Figure 6A–C). After comparison with the available standards from the GC-MS NIST library data, intermediate 1 was identified as *N*,*N*-dimethyl-benzenamine, intermediate 2 was identified as 3-dimethylamino-phenol, and intermediate 3 was identified as 4-dimethylaminobenzophenone.

#### 3.3.3. Monitoring of Intermediates after Reactive Blue 19 Degradation

**Figure 7:**
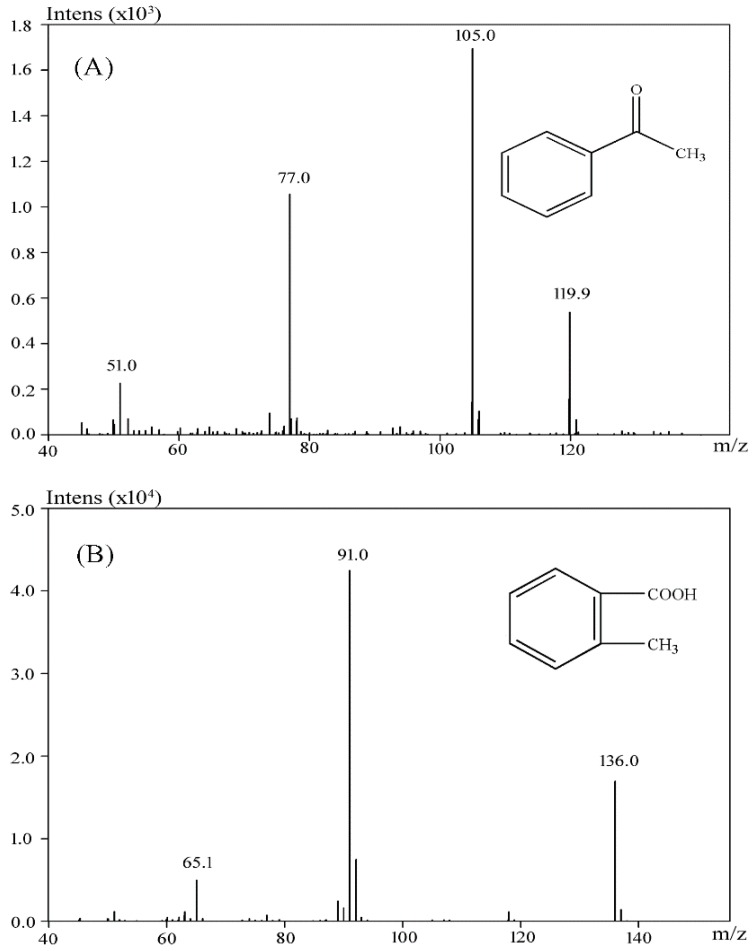
Identification of metabolites of Reactive Blue 19 by GC-MS analysis. (**A**) acetophenone (retention time 10.98 min); (**B**) 2-methylbenzoic acid (retention time 16.21 min).

The products of Reactive Blue 19 after biodegradation were assessed by GC-MS. Two products with retention times 10.98 and 16.21 were identified. After comparison with the available standards from the GC-MS NIST library data, intermediate 1 was identified as acetophenone, and intermediate 2 was identified as 2-methylbenzoic acid (Figure 7A,B). These results indicated that the anaerobic sludge is able to clave the anthraquinone ring. Notably, several other chromatographic peaks were also found but could not be positively identified. Because no additional useful intermediate could be identified, the specific pathway for Reactive Blue 19 degradation is still unclear and requires further study.

## 4. Discussion

In our study, the anaerobic sludge decolorized three types of dyes, and it presented much higher decolorization ability than that of pure bacterial strains. In general, dye treatment systems composed of mixed microbial populations have a higher efficiency of dye decolorization. Enhanced rates of decolorization of various dyes using mixed bacterial cultures have also been reported [17,18]. In mixed bacterial systems, the individual strains may attack the dye molecule at different positions or may utilize metabolites produced by the co-existing strains for further degradation. The complementary role of the different bacterial isolates in the consortium leads to an increase in decolorization efficiency. In recent years, the efficacy of various anaerobic treatment applications for the decolorization of synthetic dyes had been investigated [2]. However, most bacteria and mixed cultures can decompose only one type of dyes with similar chemical structures. There are very few examples of bacteria possessing the ability to decolorize all the azo, anthraquinone and triphenylmethane dyes in a single decolorization system. In our study, the decolorization tests indicated that the anaerobic sludge that we acclimated showed non-specificity for different dyes decolorization. Hence, the anaerobic sludge has promising potential for removing recalcitrant dye pollutants from contaminated environments.

Several studies had reported a positive relationship between the incubation time and COD removal rate. For example, Işik and Sponza have found that, in an anaerobic dye treatment reactor, the COD removal rate decreased from 79.9% to 29.4% when the incubation time was decreased from 100 to 6 h [19]. However, in our study, the COD was drastically decreased during the first 6 h incubation, and nearly unchanged in the second 6 h incubation. Similar results have also been observed by Ong et al., who found that after a 25 h incubation, the COD of the wastewater decreased from 1300 to 600 mg/L, and a longer incubation time did not produce further COD reduction [20].

Phylogenetic analysis indicated that microbial populations in the anaerobic compartment belonged to the genera *Lactococcus*, *Clostridium*, *Bacillus*, *Prevotella*, *Bacteroides*, *Longilinea* and *Klebsiella*.

*Lactococcus* is Gram-positive bacterium that generates lactic acid during growth. In recent years, the dye decolorization ability of lactic acid bacteria has been reported. You and Teng have developed an anaerobic sequencing batch reactor (SBR) that can effectively treat wastewater containing Reactive Black 5. Isolation and identification of the degrading bacteria showed that five strains belonged to different subspecies of *Lactococcus lactis* [21]. Moreover, Amani and Ahwany have found that another lactic acid bacterium, *Oenococcus oeni* ML34, can decolorize three different azo dyes [22]. All these results indicate that lactic acid bacteria have great potential for use in dye decolorization treatments.

Some species belonging to the genus *Bacillus* have been widely reported to have a strong ability to degrade different types of dyes [23,24]. Similarly to *Bacillus*, *Clostridium* sp. is wide spread in different types of anaerobic azo dye decolorization systems. Yang et al. have traced the bacteria populations in a full-scale printing and dyeing wastewater treatment system and found that, in spite of variation in the microbial community and composition of the influents, *Clostridium* sp. could be detected in all the test samples [25]. Fernando et al. have developed an anaerobic mixed culture capable of decolorizing a broad-spectrum of azo dyes. Phylogenetic analysis of 16s rDNA sequences obtained from the anaerobic culture revealed a majority was composed of bacterial species belonging to the genus *Clostridium* [26]. In addition to azo dyes, *Clostridium* sp. has proven to be effective for degradation of many triphenylmethane dyes. Kim et al. have isolated a novel strain of *Clostridium perfringens* that can decolorize a wide range of triphenylmethane dyes with a 27 h anaerobic incubation [27]. *Bacillus* and *Clostridium* are all Gram-positive bacteria that belong to the family Bacillaceae. The strong ability of the two genera to decolorize different types of dyes supports their use in the treatment of industrial wastewater containing synthetic dyes.

Little information concerning the pure isolates, which belong to Bacteroidetes, supports the potential for dye wastewater treatment. However, Zhang et al. have determined the composition and diversity of the bacterial community present in an anaerobic sequencing batch reactor used for treating dye wastewater. The results demonstrated that Bacteroidetes was the dominant phylum in the anaerobic sludge [28]. In our study, two genera belonging to Bacteroidetes were also detected in the anaerobic sludge, thus indicting that these bacterial species might play important roles in pollutant degradation or sustaining sludge particles.

Numerous studies have reported that *Klebsiella* have the ability to metabolize azo compounds [29,30]. Moreover, in our previous study, we have found that *Klebsiella* is the dominant genus in both bacterial consortia that was able to decolorize azo dyes under aerobic or anaerobic conditions [31]. In addition, many species belonging to Enterobacteriaceae, such as *Shigella*, *Enterobacter* and *Escherichia*, have proven to have dye decolorization ability [5,6,32].These reports suggest that it is likely that most species in the family Enterobacteriaceae have the potential to decolorize azo dyes.

GC-MS and LC-MS analysis demonstrated that the mechanism of microbial degradation of azo dyes by the anaerobic sludge involves the reductive cleavage of azobonds, thereby resulting in the formation of colorless solutions containing potentially aromatic amines. It was widely accepted that the first step in the bacterial degradation of azo dyes, in either anaerobic or aerobic conditions, is the reduction of the N=N bond. However, in different decolorization systems, this reduction may involve different mechanisms, such as enzymes, low molecular weight redox mediators and chemical reduction by biogenic reductants [33]. In this study, the enzyme activities of the crude cell extracts from the anaerobic sludge was studied. However, none of azoreductase activity could be detected in the cytoplasmic extracts. It indicated that the azo dye reduction in this work is not a specific reaction which is performed by azoreductase. Moreover, we observed that in our biodegradation system, the addition of synthetic electron carriers, such as lawsone and menadione, could greatly enhance the decolorization efficiency of the azo dyes. All these results showed that the azo reduction by the anaerobic sludge appeared to be nonspecific. Electrons were shuttled by various redox mediators from the bacteria to azo dyes, which leadsto the reduction of the N=N bond.

It has been reported that azo dyes cannot be mineralized completely by bacteria under anaerobic conditions [34]. After decolorization, the corresponding aromatic compounds were stably contained in the degradation system. These compounds are also harmful to the environment and needed to be further treated under aerobic conditions.

In our study, three products after Malachite Green decolorization were indentified. Based on the previous studies [35,36], possible metabolic pathway followed by the anaerobic sludge during Malachite Green degradation was proposed. At first, Malachite Green was converted to the leuco form of the dye (Leucomalachite Green). Then the leuco form product was cleaved to *N*,*N*-dimethyl-benzenamine and 4-dimethylaminobenzophenone. Then, 4-dimethylaminobenzophenone was degraded to 3-dimethylamino-phenol and benzaldehyde.

The metabolic pathway of Malachite Green proposed in this study showed some deviation from the findings of other studies. It has been reported that the first step in the biodegradation of Malachite Green is the reduction the dye to its leuco-form [37]. The Leucomalachite Green then underwent stepwise removal of its methyl groups from a single side or both sides of nitrogen [38,39]. Thus, some demethyl metabolites, such as demethylLeucomalachite Green, 4-aminobenzophenone and aniline, could be detected in the degradation systems. However, in our study, the *N*-demethylation reaction was not involved in Malachite Green degradation process. The Leucomalachite Green was hydrolyzed and then oxidized to form *N*,*N*-dimethyl-benzenamine and 4-dimethylaminobenzophenone. Then, 4-dimethylaminobenzophenone was hydrolyzed to small pieces.

## 5. Conclusions

In our study, we developed an anaerobic sludge for dye decolorization. Our results showed that the sludge decolorized various types of dyes and showed a high efficiency for continuous wastewater treatment. PCR-DGGE analysis revealed that dynamic changes in the microbial communities were occurred in the anaerobic compartments. Sequencing results indicated that the dominant members in the sludge belonged to five different phyla. The results suggested that some of the members are probably efficient degraders of dyes. Some intermediates of the three types of dye were identified, thus indicating that the sludge is able to degrade the dye molecules into smaller fragments through different mechanisms.

## Figures and Tables

**Table 1:** Azo, anthraquinone and triphenylmethane dyes used in this study.

Dyes	Chemical Structure	Tapes of Dye	λ_max_	Solubility
Methyl Orange	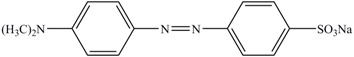	Azo	464	Water soluble
Methyl Red	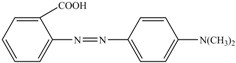	Azo	430	Alcohol soluble
Ponceau S	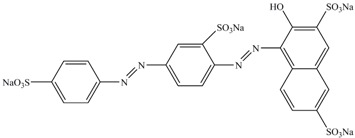	Azo	515	Water soluble
Acid red 27	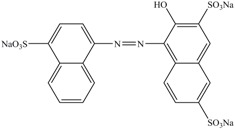	Azo	521	Water soluble
Chromotrope 2R	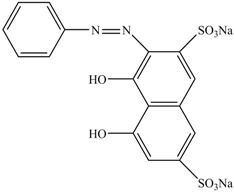	Azo	508	Water soluble
Disperse Red 11	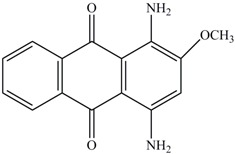	Anthraquinone	564	Water soluble
Disperse Blue 14	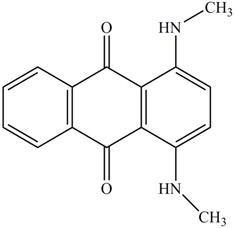	Anthraquinone	650	Alcohol soluble
Alizarin Red S	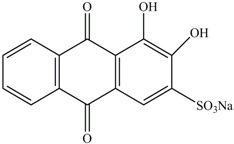	Anthraquinone	520	Water soluble
Reactive Blue 19	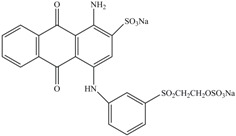	Anthraquinone	592	Water soluble
Bromoamine Acid	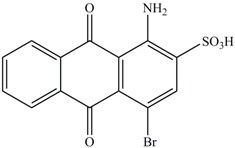	Anthraquinone	492	Water soluble
Malachite Green	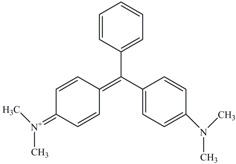	Triphenylmethane	618	Water soluble
Coomassie Brilliant Blue	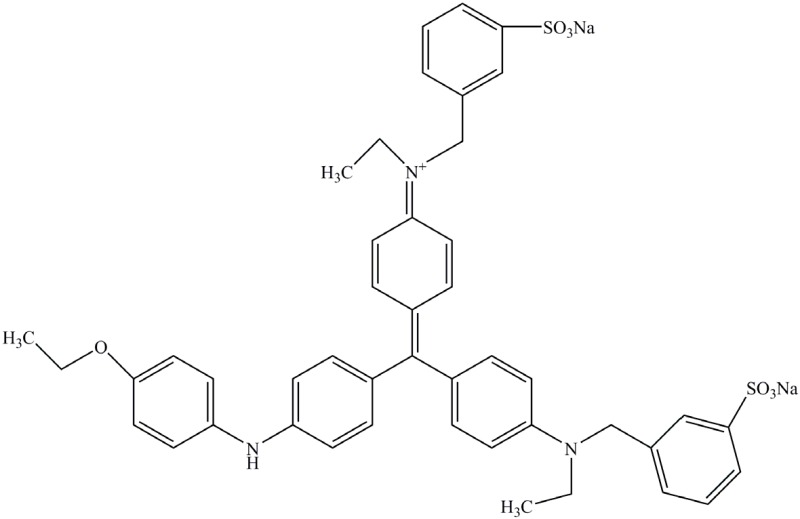	Triphenylmethane	553	Water soluble
Methyl Violet	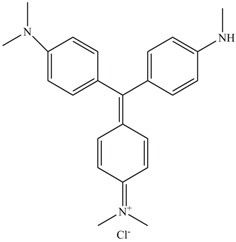	Triphenylmethane	580	Water soluble
Basic Violet 14	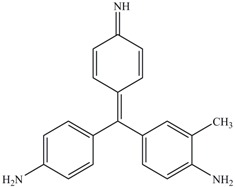	Triphenylmethane	547	Water soluble
Fast Green FCF	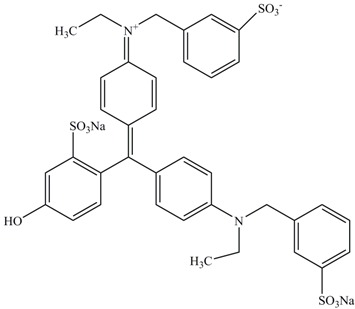	Triphenylmethane	625	Water soluble

**Table 2:** Decolorization of three types of dye by the anaerobic sludge under continuous conditions.

Dyes	Decolorization Rate (%)				
	1 Day	2 Day	3 Day	4 Day	5 Day
Methyl Orange	99.29 ± 0.67	98.16 ± 1.17	98.06 ± 1.22	96.87 ± 0.49	97.96 ± 1.12
Malachite Green	93.78 ± 1.72	93.38 ± 0.85	91.57 ± 0.93	90.86 ± 1.14	92.45 ± 1.62
Reactive Blue 19	82.15 ± 1.43	78.51 ± 0.83	79.28 ± 0.19	80.42 ± 0.74	65.23 ± 1.62
	**6 Day**	**7 Day**	**8 Day**	**9 Day**	**10 Day**
Methyl Orange	95.26 ± 0.48	96.36 ± 1.89	96.13 ± 0.63	97.02 ± 0.71	97.36 ± 0.70
Malachite Green	90.21 ± 1.32	92.45 ± 0.86	91.78 ± 1.00	90.54 ± 0.83	89.03 ± 0.96
Reactive Blue 19	68.96 ± 0.75	78.34 ± 1.87	77.54 ± 1.92	72.67 ± 1.00	74.21 ± 1.59

**Table 3:** 16S rDNA sequence analysis and species identification of selected dominant DGGE bands for the anaerobic sludge.

Band No.	GenBank No.	Sequence Length (bp)	Closest Sequences	Closest Sequences GenBank No.	Similarity (%)
AS-1	KR066383	196	Uncultured Acidobacteria bacterium clone 3E6	KC442469	100
AS-2	KR066384	194	Uncultured bacterium clone KPA8-96	KF269782	99
AS-3	KR066385	195	*Lactococcus chungangensis* strain CAU 28	NR_044357	98
AS-4	KR066386	170	*Longilinea arvoryzae* strain KOME-1	NR_041355	99
AS-5	KR066387	195	*Lactococcus* sp. R.M17	HG937722	100
AS-6	KR066388	195	*Lactococcus chungangensis* strain W1MRS32c	KF193937	100
AS-7	KR066389	189	*Prevotella paludivivens* strain JCM 13650	NR_113122	98
AS-8	KR066390	168	*Clostridium ghonii* strain NCIMB 10636	NR_119036	98
AS-9	KR066391	193	*Klebsiella* sp. H11	JN049599	99
AS-12	KR066394	189	Uncultured *Bacteroides* sp. clone L26	GQ332238	99
AS-13	KR066395	169	Uncultured bacterium clone A1-3-2	AY675970	100
AS-14	KR066396	193	*Bacillus cohnii* strain NBRC 15565	NR_113776	99
AS-15	KR066397	194	Uncultured bacterium clone K119	AM418664	100
AS-16	KR066398	169	Uncultured bacterium clone D5Day21bio2cold	GU171141	97
